# Implementation of the 2022 AAP guidelines for neonatal hyperbilirubinemia could reduce the need for phototherapy in Italy

**DOI:** 10.1186/s13052-025-02002-x

**Published:** 2025-05-28

**Authors:** Carlo Dani, Monica Fusco, Marco Andreini, Giuseppe Pepe, Simone Pratesi

**Affiliations:** 1 Department of Neurosciences, Psychology, Drug Research, and Child Health, University of Florence, Florence, Italy; 2 Division of Neonatology, Careggi University Hospital of Florence, Largo Brambilla 3, Firenze, 50134 Italy

**Keywords:** Hyperbilirubinemia, Phototherapy, Guidelines, Late preterm, Term, Infant

## Abstract

**Background:**

The American Academy of Pediatrics (AAP) revised in 2022 its guideline on the management of neonatal hyperbilirubinemia and suggested a significant increase in the thresholds for phototherapy. Our aim was to evaluate if the implementation of these guideline could reduce admissions for hyperbilirubinemia requiring phototherapy in our unit.

**Methods:**

We studied 876 infants with gestational age *≥* 35 weeks who were admitted for hyperbilirubinemia requiring phototherapy during the first week of life. Total serum bilirubin (TSB) at the start of phototherapy, which was decided based on the guidelines of the Italian Society of Neonatology, was compared with the TSB thresholds recommended by AAP 2022 guidelines.

**Results:**

Seven hundred and thirteen (82%) infants had TSB at the start of phototherapy lower than AAP 2022 threshold (16.2 *±* 3.0 vs. 17.7 *±* 3.4 mg/dL; *P* < 0.001) with a mean difference of 1.8 (0.7–2.6) mg/dL. Among them, one hundred and fifteen infants (13%), 226 (26%), and 372 (42%) had TSB slightly (0.1-1-0 mg/dL), moderately (1.1-2.0 mg/dL), or greatly (> 2.0 mg/dL) below AAP threshold.

**Conclusions:**

It can be estimated that the implementation of the AAP 2022 guidelines in our unit could reduce the rate of hospitalizations for hyperbilirubinemia requiring phototherapy by 42 to 68%. These findings, along with the short- and long-term neonatal and economic benefits, support the implementation of the AAP 2022 guidelines in our unit.

## Background

A high level of total serum bilirubin (TSB) can be neurotoxic to the central nervous system in term and preterm infants, causing acute bilirubin encephalopathy (ABE) and neurological kernicterus spectrum disorders (KSDs) 1. Therefore, recommendations have been made for preventing ABE and KSDs in the neonatal period by establishing TSB threshold values ​​at which to start phototherapy, the first-choice treatment of neonatal hyperbilirubinemia. Some national guidelines also indicate the TSB threshold values ​​at which to start phototherapy in preterm infants of different gestational ages 2, 3.

However, the assumption that this therapy is innocuous with no serious adverse effects for even the most immature babies has been questioned. In fact, a randomized trial demonstrated that aggressive phototherapy increases the mortality in extremely preterm infants 4, and other studies reported several adverse effects, such as photo-oxidative injury, lipid peroxidation, DNA damage, reduced splanchnic oxygenation, feeding intolerance, and hemolysis 5, 6, 7, 8, 9, 10, 11, 12, 13, 14, 15, 16, 17, 18. Moreover, it has been suggested that these adverse effects of PT might increase the risk of epilepsy 19, 20 and cancer 21, 22, 23.

Therefore, it is important that further studies 24, 25, 26 suggested that phototherapy can be safely initiated at significantly higher levels of TSB reducing the number of treated infants. Consistently, the American Academy of Pediatrics (AAP) revised its guidelines on the management of neonatal hyperbilirubinemia including, among other key recommendations, a significant increase in TSB thresholds for phototherapy and exchange transfusion 27. Moreover, the guideline suggested that it may be appropriate for clinicians take into account individual circumstances when assessing patients’ risk factors and deciding when to initiate phototherapy 27. Indeed, as expected, Sarathy et al. 28 and Michienzi et al. 29 reported that the implementation of these updated guidelines 27 in their hospitals was followed by a significant reduction in the use of phototherapy and TSB measurements. This decreased hospitalization of newborns and their separation from mothers with evident socioeconomic and health benefits.

Based on these considerations, we hypothesized that using AAP 2022 ^27^ thresholds to start phototherapy instead of those suggested by the Italian Society of Neonatology 2 could reduce hospital admissions for hyperbilirubinemia. To evaluate this hypothesis and, preliminary to the possible implementation of the AAP 2022 guidelines 27 in our unit, we performed this retrospective study that assessed how many neonates admitted to our special care unit for hyperbilirubinemia requiring phototherapy would have been admitted using the AAP 2022 thresholds 27.

## Methods

The study was carried out at the Neonatal Special Care Unit of the Careggi University Hospital of Florence, after approval by the local ethics committee. Infants with gestational age *≥* 35 weeks were eligible for the study if they were in good clinical condition and presented hyperbilirubinemia requiring phototherapy during the first week of life. Exclusion criteria were congenital infections.

### Study design

The value of TSB at the start of phototherapy was compared with the TSB threshold recommended by AAP 2022 guidelines 27.

Entry criteria to phototherapy followed the recommendations of the Italian Society of Neonatology 2, and the decision to start treatment was made by the attending neonatologist. Blue light emitting diodes was placed about 30 cm above the infant, as recommended by the manufacturers, and phototherapy was discontinued when TSB levels were below the threshold value for treatment on two consecutive measurements 6 and 12 h after the start of treatment 2.

TSB was measured in whole blood from a heel prick sample in a blood gas analyzer 30 with a spectrophotometer module (ABL 735™, Radiometer, Fiske Street Holliston, MA, USA).

The daily care (i.e.: feeding, vital sign monitoring, etc.) of enrolled infants was carried out according to local protocols.

### Other collected data

We recorded the following data for each infant: gestational age, birth weight, twinship, sex, type of delivery, ABO and Rh mismatch, positive direct antibody test (DAT), G6PD deficiency, transcutaneous bilirubin (TcB) measurements followed by TSB measurements, age and TSB value at the start of phototherapy, phototherapy duration, treatment with i.v. immunoglobulins and exchange transfusion, readmission for phototherapy, and lengths of stay in hospital.

### Statistical analysis

The primary endpoint of our study was to assess how many infants started phototherapy with a TSB value below the AAP 2022 thresholds 27. Secondary endpoint was to evaluate the differences between TSB measured at start of phototherapy and AAP 2022 TSB thresholds 27. To distinguish infants with a slight, moderate, and great difference between TSB at start of phototherapy and AAP thresholds 27, we calculated how many infants had TSB values 0.1-1.0, 1.1-2.0 mg/dL, or > 2.0 mg/dL below the AAP threshold, respectively 27.

Data are reported as mean values and standard deviations, median values and interquartile ranges, or rates and percentages. Comparisons were performed using the Student’s t test for parametric continuous variables with 80% power at 0.05 level.

## Results

Eight hundred and seventy-six infants were consecutively enrolled in the study from December 2017 to July 2024. The admission rate for hyperbilirubinemia requiring phototherapy was 4.2% (876/21.031). Clinical characteristics of studied infants are reported in Table 1.

**Table 1:** Demographic and clinical characteristics of studied infants. Mean *±* (SD), rate and (%), median and (IQR)

	*N* = 876
Gestational age (wks) <38 wks	38.3 *±* 1.5 265 (30)
Birth weight (g) <10° percentile	3197 *±* 489 70 (8)
Twins	20 ((2)
Female	357 (41)
Cesarean section	121 (14)
ABO blood type mismatch	123 (14)
Rh mismatch	128 (15)
Positive Direct Antibody Test	164 (19)
Glucose-6-phosphate dehydrogenase deficiency	5 (0.6)
Other hemolytic diseases	4 (0.5)
TcB measurements TcB measurements followed by TSB control Values of TcB (mg/dL)	229 (26) 222 (25) 13.6 *±* 2.5
Age at the start of phototherapy (h)	79.0 *±* 40.8
TSB at the start of phototherapy (mg/dL)	16.2 *±* 3.0
ISN threshold for phototherapy AAP threshold for phototherapy Median difference (mg/dL)	16.4 *±* 3.0 17.7 *±* 3.4 1.6 (0.9–2.1)
TSB at start of phototherapy below AAP threshold Median difference (mg/dL)	713 (82) 1.8 (0.7–2.6)
TSB at start of phototherapy 0.1-1.0 mg/dL below AAP threshold	115 (13)
TSB at start of phototherapy 1.1-2.0 mg/dL below AAP threshold	226 (26)
TSB at start of phototherapy > 2.0 mg/dL below AAP threshold	372 (42)
Phototherapy duration (h)	22.9 *±* 11.7
Intravenous immunoglobulin	17 (5)
Exchange transfusion	0
Readmission for phototherapy	160 (18)
Length of stay (h)	133 *±* 95

TcB: transcutaneous bilirubin; TSB: total serum bilirubin; ISN: Italian Society of Neonatology; AAP: American Academy of Pediatrics

ABO, Rh mismatch, and G6PD deficiency were found in 123 (14%), 128 (15%), and 164 (19) infants, respectively. Positive DAT and other hemolytic diseases were detected in 5 (0.6%) and 4 (0.5%) patients, respectively. TcB assessment preceded TSB measurement in 222 (25%) patients.

**Fig. 1:**
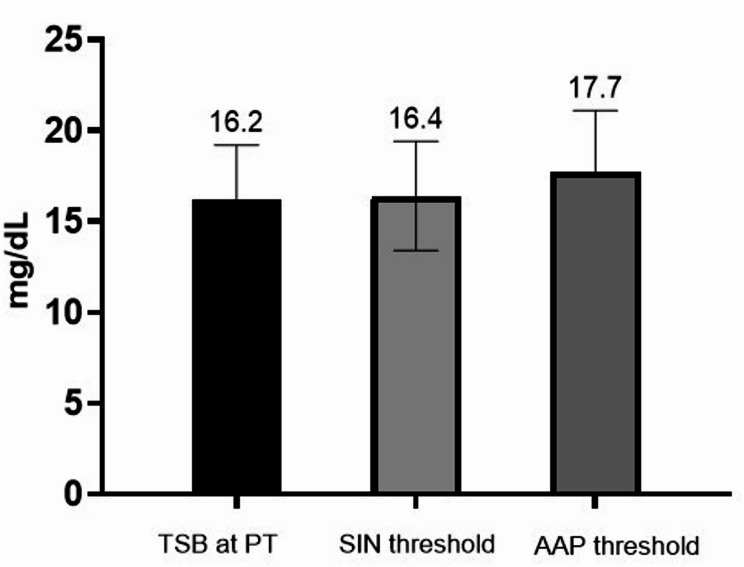
Comparison between total serum bilirubin (TSB) measured at the start of phototherapy and the threshold suggested by the Italian Society of Neonatology 2 and the American Academy of Pediatrics 27

Phototherapy was started at 79.0 *±* 40.8 h of life at a mean TSB value similar to the threshold of the Italian Society of Neonatology 2 (16.2 *±* 3.0 vs. 16.4 *±* 3.0 mg/dL; *P* = 0.163) and lower than that of the AAP 2022 guidelines 27 (16.2 *±* 3.0 vs. 17.7 *±* 3.4 mg/dL; *P* < 0.001). (Fig. 1)

Seven hundred and thirteen (82%) infants had TSB at the start of phototherapy lower than AAP threshold 27 with a median difference of 1.8 (0.7–2.6) mg/dL.

**Fig. 2:**
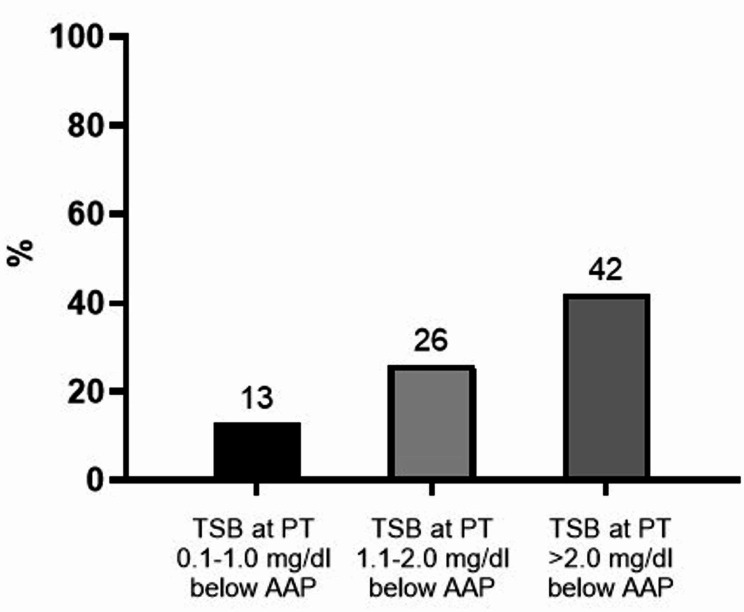
Percentage of neonates with total serum bilirubin (TSB) slightly (0.1-1.0 mg/dL), moderately (1.1-2.0 mg/dL), or greatly (> 2.0 mg/dL) below the AAP threshold 27

Among them, 115 (13%), 226 (26%), and 372 (42%) infants had TSB slightly (0.1-1-0 mg/dL), moderately (1.1-2.0 mg/dL), or greatly (> 2.0 mg/dL) below AAP threshold 27, respectively. (Fig. 2).

Length of stay in hospital was 133 *±* 95 h and the readmission rate for hyperbilirubinemia was 18% (*n* = 160).

## Discussion

In this study we evaluated how many neonates admitted to our special care unit for hyperbilirubinemia requiring phototherapy would be admitted using the AAP 2022 thresholds 27. We found that 82% of studied infants had TSB below AAP threshold 27 and that this difference was moderate and great in 26 and 42% of cases, respectively. Therefore, assuming that the application of the AAP 2022 ^27^ guidelines can avoid hospital admissions for hyperbilirubinemia requiring phototherapy in the majority of these two categories of patients, a reduction in hospitalizations of 42 to 68% can be estimated.

Our results agreed with Sarathy et al. who studied more than 22.000 infants with gestational age *≥* 35 weeks and found that the application of AAP 2022 guidelines 27 instead of those of AAP 2004 ^3^ was followed by a 47% decrease in the phototherapy admission rate, from 3.9 to 2.1%, with no change in the phototherapy readmission rate (0.9 vs. 0.8%) 28. Similarly, Michienzi et al. found that the implementation of AAP 2022 guidelines 27 decreased the need for phototherapy, from 4.2 to 1.4% with no changes in readmission rate 29. It is of note that Sarathy et al. (3.9%) 28 and Michienzi et al. (4.1%) 29 reported an initial admission rate for phototherapy like ours (4.2%) and this supports the transferability of their results to our population.

The decrease in admission rate for hyperbilirubinemia requiring phototherapy can have several short- and long-term advantages. Among the former, to reduce unnecessary neonatal admissions contributes to limit the infant-family separation which can lead to parental stress and anxiety and hampers the establishment of exclusive breastfeeding 31. This approach is, therefore, consistent with the goal of zero separation for all newborns from their family which should also be pursued adopting therapeutic strategies which allow to safely minimize admission in neonatal care units 32. Moreover, increased thresholds for starting phototherapy can decrease the need for painful TSB measurements, as found by Sarathy et al. 28 and Michienzi et al. 29 who reported a decrease of 22 and 13%, respectively. Regarding long-term advantages, Maimburg et al. reported that male infants treated with phototherapy for hyperbilirubinemia had a higher risk of developing epilepsy in early childhood 19 and these results were confirmed by Newman et al. 20. Wickremasinghe et al. found a slight increased risk of overall cancer, myeloid leukemia, and kidney cancer after phototherapy with a number needed to harm of 10.638 ^21^. Newman et al. reported an association between phototherapy use and increased rates of cancer (particularly nonlymphocytic leukemia), although controlling for confounding variables eliminated or attenuated these associations 22. In any case, these risks must be considered when making decisions about phototherapy treatment and when deciding on the adoption of guidelines that affect phototherapy thresholds.

A further benefit of the application of guidelines reducing the need of neonatal admission is of economic nature. The burden of resources required to treat neonatal hyperbilirubinemia is very high, as it causes the 5% of neonatal admission from within the hospital and is the most common reason for post discharge re-admission (22%) 33. Therefore, reducing hospitalizations for phototherapy can help optimize care costs and free up resources for other tasks.

Limitations of our study include its retrospective design, but the data presented reflect care provided at a single Italian III level neonatal care unit for more than 6-years in a homogeneous population. We are therefore confident that our results are accurate and reproducible in other centers where current guidelines on the management of neonatal hyperbilirubinemia suggest higher thresholds for phototherapy than those of the AAP 2022 ^27^.

## Conclusions

We found that 82% of studied infants had TSB at the start of phototherapy below AAP threshold 27 and that this difference was moderate or great in 26 and 42% of cases, respectively. Therefore, it can be estimated that the implementation of the AAP 2022 guidelines 27 in our unit could reduce the rate of hospitalizations for hyperbilirubinemia requiring phototherapy by 42 to 68%. These findings, along with the short- and long-term neonatal and economic benefits, support the implementation of the 2022 AAP guidelines 27 in our unit.

## Data Availability

The datasets used and/or analysed during the current study are available from the corresponding author on reasonable request.

## Abbreviations

AAP: American Academy of Pediatrics
SIN: Società Italiana di Neonatologia
TcB: Transcutaneous bilirubin
TSB: Total serum bilirubin
